# Patient capacity and constraints in the experience of chronic disease: a qualitative systematic review and thematic synthesis

**DOI:** 10.1186/s12875-016-0525-9

**Published:** 2016-09-01

**Authors:** Kasey R. Boehmer, Michael R. Gionfriddo, Rene Rodriguez-Gutierrez, Abd Moain Abu Dabrh, Aaron L. Leppin, Ian Hargraves, Carl R. May, Nathan D. Shippee, Ana Castaneda-Guarderas, Claudia Zeballos Palacios, Pavithra Bora, Patricia Erwin, Victor M. Montori

**Affiliations:** 1 Knowledge and Evaluation Research (KER) Unit, Mayo Clinic, 200 First Street SW, Rochester, MN 55905 USA; 2 Mayo Graduate School, Mayo Clinic, Rochester, MN USA; 3 Endocrinology Division, University Hospital “Dr. Jose E. Gonzalez”, Universidad Autonoma de Nuevo Leon, Monterrey, Mexico; 4 University of Southampton, School of Health Sciences, Southampton, UK; 5 Division of Health Policy and Management, School of Public Health, University of Minnesota, Minneapolis, MN USA; 6 Department of Emergency Medicine, Mayo Clinic, Rochester, MN USA; 7 Mayo Medical Libraries, Mayo Clinic, Rochester, MN USA

## Abstract

**Background:**

Life and healthcare demand work from patients, more so from patients living with multimorbidity. Patients must respond by mobilizing available abilities and resources, their so-called capacity. We sought to summarize accounts of challenges that reduce patient capacity to access or use healthcare or to enact self-care while carrying out their lives.

**Methods:**

We conducted a systematic review and synthesis of the qualitative literature published since 2000 identifying from MEDLINE, EMBASE, Psychinfo, and CINAHL and retrieving selected abstracts for full text assessment for inclusion. After assessing their methodological rigor, we coded their results using a thematic synthesis approach.

**Results:**

The 110 reports selected, when synthesized, showed that patient capacity is an accomplishment of interaction with (1) the process of rewriting their biographies and making meaningful lives in the face of chronic condition(s); (2) the mobilization of resources; (3) healthcare and self-care tasks, particularly, the cognitive, emotional, and experiential results of accomplishing these tasks despite competing priorities; (4) their social networks; and (5) their environment, particularly when they encountered kindness or empathy about their condition and a feasible treatment plan.

**Conclusion:**

Patient capacity is a complex and dynamic construct that exceeds “resources” alone. Additional work needs to translate this emerging theory into useful practice for which we propose a clinical mnemonic (BREWS) and the ICAN Discussion Aid.

**Electronic supplementary material:**

The online version of this article (doi:10.1186/s12875-016-0525-9) contains supplementary material, which is available to authorized users.

## Background

Patient capacity has been defined as the available abilities and resources a patient can mobilize to address the demands healthcare and life make. Limitations in capacity impact a patient’s “ability or readiness to do work” [1]. Patients with multiple chronic conditions and their caregivers may face challenges in meeting the demands of both self-care and healthcare. Characterizing the role that capacity plays in this effort has become an important area of investigation [2–5]. Insights to date suggest that a key and distinguishing aspect of capacity is that it is distributed amongst many life activities and linked to the social networks of patients. Specifically, the capacity patients must use to meet demands in health, i.e., to face the burden of treatment, is the same set of abilities and resources that they use to meet obligations in life and to fulfill the roles that bring meaning to it.

In prior research and through clinical experience, we developed a working list of six domains of patient capacity: Personal, Physical, Mental, Social, Financial, and Environmental [6]. Mental and physical capacity relate to health and are limited by the “burden of illness:” either by the disease (i.e., cognitive dysfunction due to heart failure, physical function limitations from arthritis) or its treatment (i.e., side effects from chemotherapy or dialysis). Personal, social, financial, and environmental capacity may be limited by scarcity: patients may be stressed or burnt out, lack adequate literacy, suffer from isolation, live in poverty, or be at a distance from healthcare and social support.

When patient capacity is inadequate to shoulder the work of healthcare and life, patients may not be able to access to and use of healthcare and the potential for self-care. This, in turn, can have a negative effect on health outcomes [1]. Still, patients may be able to draw upon available capacity in some domains in order to overcome limitations in others. They may also report surprisingly low disruption from illness and treatment despite high levels of healthcare work [3]. This suggests a dynamic relationship between elements of patients’ capacity that makes it on the whole difficult to define, measure, or discuss in clinical practice. Capacity may exist beyond easily categorized domains of resources, instead consisting of both objective and subjective elements, which serve different purposes in the illness journey: the capacity to survive, to cope, and to thrive.

While strides have been made to characterize the patient’s healthcare workload and how it may manifest as burdensome [4], and to create a measure of the burden of treatment [7, 8], a comprehensive and useful view of patient capacity that can be used by patients and clinicians for clinical encounter decision making is lacking. Additionally, all previous capacity domains were based in clinical and research experience, and therefore, a definition of capacity grounded in the patient experience is also missing.

### Aims

The aim of this review was to summarize the literature on patient experiences that illustrate patient capacity to access and use healthcare or enact self-care while carrying out their lives. Our practical goal was to inform the development of a discussion aid for the clinical encounter that could create a conversation among patients with one or more chronic conditions, their caregivers, clinicians, and the healthcare team around these issues (the ICAN Discussion Aid) [9]. By critically thinking about the state of the patient’s capacity, clinicians and other health professionals have a unique opportunity to partner with patients to develop and modify treatment plans that are respectful of patient capacity. This review was not intended to provide an exhaustive list of capacity that can be activated or mobilized, but rather its synthesis illuminates, a descriptive theory; [10] it names the practical considerations of patients’ capacity, both objective and subjective, beyond the existence of resources that are worthy of attention between patients with chronic conditions, their caregivers, and healthcare teams working together.

## Methods

The conduct of this systematic review followed a rigorous protocol and this report adheres to the ENTREQ statement [11].

### Study identification

To develop our search strategy, we followed methods described by Gallacher et al. for conducting a qualitative systematic review of a novel construct [12]. We first conducted scoping searches of the qualitative literature in order to pick up key articles that fit our inclusion criteria; this process helped to identify studies and key terms. As described previously by Gallacher et al., the scoping search includes a ‘berry picking’ , process of discovering groups of studies together through a preliminary search of databases, use of the ‘related articles’ function in PubMed, and consultation with experts in the field [12].

A comprehensive search of four databases (Ovid MEDLINE, Ovid EMBASE, Ovid PsycInfo, and EBSCO CINAHL) published from January 2000 to May 2014 was conducted. An experienced librarian (PE) designed the search strategy with input from study investigators (KB and VMM) with expertise in conducting systematic reviews. Controlled vocabulary supplemented with keywords was used to search for studies that described limiters of capacity or barriers that patients with chronic conditions experience in their lives. The search strategy is available in the Additional file 1.

### Eligibility criteria

We included in-depth interviews, focus groups, or ethnographic studies in which limiters of capacity or barriers that patients with chronic conditions experienced in their lives as part of accessing and using healthcare or enacting self-care were described. We sought studies with qualitative methods because we aimed to synthesize rich descriptions of patient capacity. Chronic conditions were defined as: a condition “that lasts 12 months or more and either limits self-care or independent living or requires ongoing medical intervention” [13]. Access and use included both the availability of the services as well as the ability to realize the use of available services when it was needed or desired [14]. We used the definition of self-care activities by Bayliss, et al.: activities that patients did to 1) promote their physical and psychological health, 2) engage with healthcare providers and maintain adherence to recommended treatments, 3) monitor their health status and make associated healthcare decisions, or 4) manage the impact of their illness(es) on physical, psychological, or social functioning [15]. Barriers were defined as any part of the patient’s life or healthcare that delayed, prevented, or minimized their ability to access or use healthcare or to enact self-care.

Studies were excluded if their primary unit of analysis was not the patient (i.e., patient-provider team, family, caregiver as a surrogate for the patient). We excluded studies that used strictly quantitative methodology to answer their research question. Studies that were mixed methods were included if their results placed sufficient priority on the qualitative patient experience. We made this determination by applying Creswell’s criteria of priority in mixed methods studies: “noting the relative emphasis given to framing the research problem (e.g., intent to test a theory, study variables, or explore constructs) or the subservient use of 1 form of data to the other (e.g., qualitative data helps to build an instrument).” [16] Mixed methods studies that gave priority to quantitative design and reporting, particularly following the “instrument design model” or the “data transformation model” were excluded, due to their lack of rich qualitative data for the synthesis [16].

### Selection of studies

Studies were screened in two stages: abstract screening and full text screening. In each of these stages, each study was screened independently and in duplicate by the lead author (KB) and one other reviewer (MG, AL, AC, CZ, PB). The chance-adjusted inter-reviewer agreement calculated using k statistic was 0.69 at the abstract screening level and 0.74 at full text screening. Disagreements were resolved through discussion and consensus among the two reviewers. When consensus could not be reached between the two reviewers, studies were sent to a third reviewer for the final decision.

### Data extraction and quality assessment

Descriptive data (time point in the patients’ disease trajectory the data were collected, research question, theoretical frameworks used, sampling procedures, data collection method, data analysis method, overall conclusion of the study, limitations, and conflicts of interest) of the included studies were extracted using Distiller SR (EvidencePartners, Ottawa, Canada). During data extraction the lead author (KB) and co-author (MG) individually and in duplicate conducted a quality assessment of each study using the Critical Appraisal Skills Programme (CASP) Qualitative Research Checklist [17]. The 9-item assessment tool asks that reviewers rate each aspect of quality (i.e., was the data collected in a way that addressed the research issue?) as “yes”, “no”, or “can’t tell” for cases in which not enough information is reported. Each question has guiding points to consider in making the appraisal of that domain. All disagreements were discussed between the two authors until consensus was reached.

### Data analysis

After data extraction, full-text manuscripts were imported into Nvivo 10 (QSR International, Burlington, MA, USA). We conducted a thematic synthesis of the results sections [18], resulting in the proposed theory of patient capacity. Three reviewers (KB, RRG, MA) coded five studies line by line to create the initial list of codes, then met to discuss and refine. The same reviewers then coded in duplicate an additional three studies using the previously generated list and taking note to identify any new themes emerging from the data. The team then met again to compare codes and agree on a final coding list. No new codes were added after this point. The lead author (KB) then analyzed the inductive themes, beginning with the code of “patient important outcomes related to using healthcare and enacting self-care”. Constructs generated from this list were further unpacked. Analysis continued by using matrices to explore overlapping concepts and to finalize the constructs of the proposed theory.

While we sought to incorporate the timeline relative to the time since diagnosis as another concept of patient experiences, there was a paucity of studies that reported this information. Where it was reported, it did not seem to inform the study design or analysis which made it impossible to include in the final analysis.

## Results

### Identification of studies

**Fig. 1:**
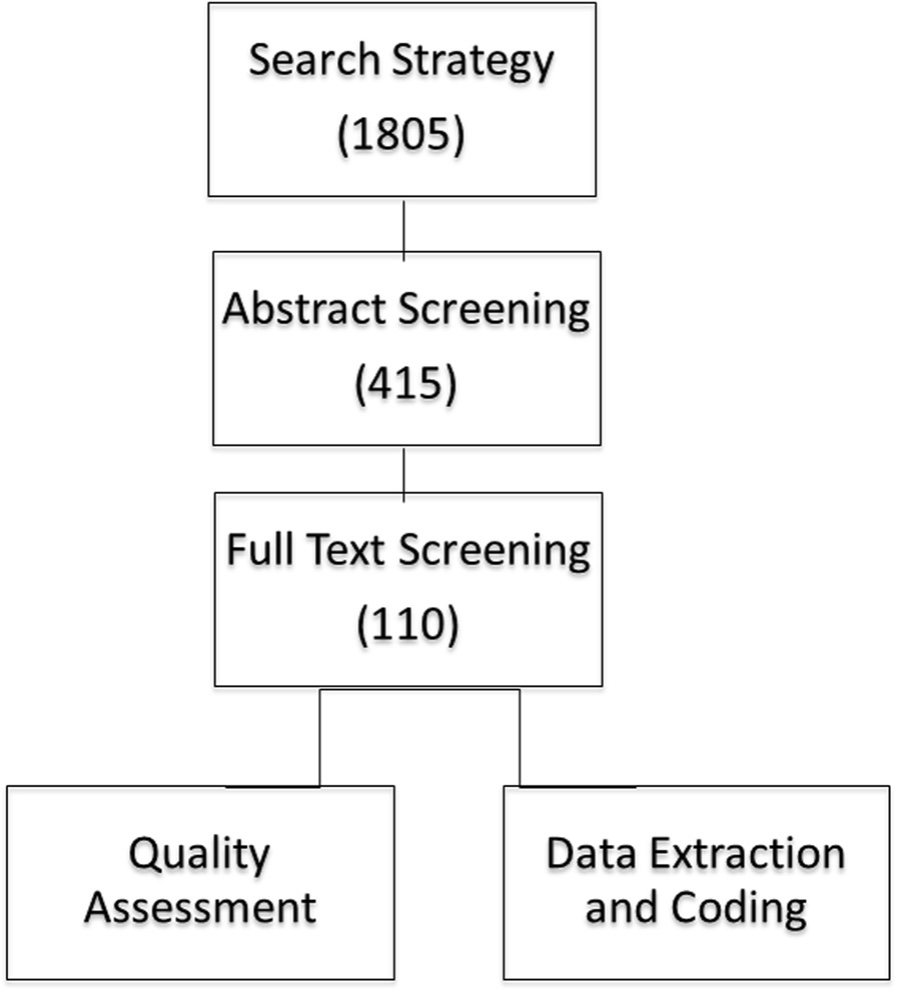
Study selection process

Our initial search strategy yielded 1805 manuscripts. After abstract screening, 405 manuscripts were moved to full-text screening. After full-text screening, we had 110 manuscripts, which were ultimately included in the study for quality appraisal, data extraction, and coding. Figure 1 depicts the study selection process.

### Summary of included studies

Table 1 describes the Included studies, mostly from the US (48) and UK (19), with a minority from elsewhere: Canada (9), Sweden (6), Norway (3), Australia (2), Iceland (1), Dominican Republic (1), New Zealand (1), and Belgium (1). Table 2 describes the methodological rigor of the included studies, which in general was good, limited by incomplete justification of the methods chosen in some and of incomplete methods reporting in a handful.

**Table 1:** Description of included studies

Author	Year	Country	Condition(s)	Theoretical framework	Sampling procedure	Data collection	Data analysis
Aspin	2012	Australia	Chronic conditions - at least one index condition of diabetes, COPD, or CHF	None Reported	Purposive Sampling	Semi-structured interviews	Content Analysis
Audulv	2013	Sweden	Ischemic heart disease rheumatic disease, chronic renal disease, inflammatory bowel disease, multiple sclerosis and diabetes	None Reported	Purposive	Semi-Structured Interviews.	Interpretive Descriptive Analysis
Bair	2009	USA	comorbid chronic musculoskeletal pain and depression	None Reported	Random Sampling of randomized control trial participants	Focus Groups	Thematic Analysis
Barker	2006	Canada	Stroke	The International Classification of Functioning, Disability and Health (ICF)(World Health Organization [WHO], 2001) and Continuity Theory	Sub-Sampling from lareger study	In-depth interviews	Constant comparative analysis
Bayliss	2008	USA	Multimorbidity - at a minimum, the combined conditions of diabetes, depression and osteoarthritis	None Reported	Randomly selected from larger survey Sampling of patients that met inclusion critieria	Semi-Structured Interviews	Thematic Analysis and Constant Comparison
Beauregard	2005	Canada	HIV/AIDS	None Reported	Convenience Sampling	Interviews	Phenomenologic analysis
Becker	2004	USA	All chronic conditions; most common diabetes, asthma, heart disease and hypertension	None Reported	Purposive Sampling	3 in-depth Interviews over a one year period	Krieger Methodology and Narrative Analysis
Becker	2003	USA	Multiple conditions; most common diabetes, asthma, heart disease, or hypertension	None Reported	Convenience Sampling	3 in-depth interviews over 1 year	Content Analysis Stratified by Income Category
Becker	2003	USA	Chronic disease in general; most common diabetes, asthma, and heart disease or hypertension	None Reported	Convenience Sampling	3 in-depth interviews over 1 year	Content Analysis and Case-by-Case Narrative Analysis
Beverly	2011	USA	Type 2 Diabetes with comorbid conditions	None Reported	Intensity (Purposive) Sampling	Focus Groups	Thematic Analysis
Boeckxstaens	2012	Belgium	COPD	None Reported	Convenience Sampling	Interviews	Thematic Analysis
Bova	2010	USA	Hepatitis C AND HIV	None Reported	Purposive Sampling and Theoretical Sampling	Semi-structured face-to-face interviews	Qualitative Descriptive Methods and Content Analysis
Bower	2012	UK	Multimorbidity (Diabetes, COPD, CHD, arthritis and depression, cancer, thyroid disease, hypertension)	Common sense model of illness	Purposive Sampling	Semi-Structured Interviews	Framework Analysis - Constant Comparison
Bremander	2009	Sweden	Chronic pain	None Reported	Patients who completed a pain rehabilitation program	Interview	Grounded Theory
Burles	2013	Canada	Anorexia, breast cancer, depression, endometriosis, epilepsy, multiple sclerosis, primary hypoadrenalism and secondary hypothyroidism, and a malignant brain tumour	None Reported	Snowball Sampling	Interpretive, Hermeneutic Phenomenological Interviews and Photovoice	Holistic and Cross-Sectional Data Analysis Guided by Hermeneutical Phenomenolgy
Carey	2005	USA	Insomnia	None Reported	Purposive Sampling	Focus Groups	Survey Development/Thematic Analysis
Clarke	2008	Canada	Multiple Chronic Conditions	Symbolic interactionism	Purposive Sampling	In-depth interviews	Grounded Theory
Conrad	2006	Australia	Chronic hepatitis C	None Reported	Purposive; snowball	Semi-structured interviews and focus groups	Grounded Theory
Corsner	2011	USA	MCC (≥2: Diabetes, Chronic Dulmonary Disease (i.e., asthma, COPD, emphysema), CHF, coronary artery disease, osteoarthritis, musculoskeletal disorder, and/or ongoing cancer/neoplasm	None Reported	Convenience Sampling	Focus Groups (supplemented with chart reviews)	Content Analysis
Coty	2013	USA	Rheumatoid Arthritis	Self-regulation Theory	Purposive Sampling of patients from larger quantitative study	Semi-Structured Telephone Interviews	Phenomenological Analysis (Colaizzi)
Drew	2006	USA	Chronic Lyme Disease	None Reported	Purposive Sampling	In-Depth Interview	Colazzi Phenomenology
Edmonds	2007	UK	Multiple sclerosis	None Reported	Purposive Sampling	Semi-structured interview	Constant comparison
Elliott	2007	USA	Multiple Chronic Conditions	None Reported	Purposive Sampling	Semi-Structured Interviews	Constant Comparison
Ellis	2013	UK	Cancer	Not Reported	Patient attending a hospice day care service was recruited (via hospice staff) and asked to invite their family members to be involved in the research	Repeat, in-depth interviews and participant observation on a hospice inpatient ward	Thematic Analysis
Eton	2012	USA	Multiple Chronic Conditions	Normalization Process Theory informed the interview guide - no theoretical framework guided the analysis	Convenience Sampling of patients already participating in a medication therapy management program	Semi-Structured Interviews	Ritchie and Lewis’ framework Analysis
Feldman	2003	USA	Arthritis	Ecological Framework	Convenience Sampling	Peer support groups	Not Reported
Fisher	2007	USA	Chronic Pain	None Reported	Purposive Sampling	Semi-Structured Interviews	Thematic Analysis
Gallant	2007	USA	Arthritis, diabetes, and/or heart disease	Social cognitive theory and others	Purposive Sampling	Focus Groups	Thematic Analysis
Gelling	2009	UK	Idiopathic normal pressure hydrocephalus	Berger and Luckmann	Purposive and Theoretical Sampling	Semi-structured interviews and written personal biographies	Grounded Theory
Gustafsson	2012	Australia	Stroke	None Reported	Single case study	E-mail conversations	Narrative Analysis
Hodgson	2011	UK	Severe and Enduring Mental Illness	None Reported	Purposive Sampling	One-to-one interviews	Thematic Analysis
Jakobsen	2001	Norway	Any Chronic Condition. Included: rheumatoid arthritis, fibromyalgia, ankylosing spondylitis, lupus, heart disease, and lower back pain.	None Reported	Purposive Sampling	Interviews and observations of workplaces	Phenomenological Analysis
Janevic	2014	USA	Asthma AND Type 2 diabetes, heart disease or arthritis requiring daily medication for at least 1 year, or report a significant effect of arthritis on daily functioning	None overall, but, the frameworks of social support and resilience were called upon to inform the study	Quota Sampling	In-Person Semi-Structured Interview	General Inductive Approach
Janke	2012	USA	Chronic Pain in Obesity	None Reported	Purposive Sampling	Individual or small group interviews	Constant Comparative Method
Janke	2008	USA	Hepatitis C	None Reported	Convenience Sampling	Focus Groups	Grounded Theory
Jeon	2012	Australia	Multiple chronic conditions	Explanatory Model of Illness	Purposive criteria selected from a previous survey	Interview by phone	Content Analysis
Jeon	2010	Australia	Type 2 Diabetes, Chronic heart failure, chronic obstructive pulmonary disease	Explanatory Model of Illness	Purposive Sampling	Semi-structured in-depth interviews	Content Analysis
Jeon	2009	Australia	Type 2 Diabetes, Chronic Heart Failure, Chronic Obstructive Pulmonary Disease	None Reported	Purposive for patients; convenience for carers; recruited through referrals	Semi-structured, in-depth interviews	Content Analysis
Jerant	2005	USA	Arthritis, asthma, COPD, CHF, depression, and DM	None Reported	Convenience Sampling	Focus Groups	Grounded Theory
Jones	2012	USA	Heart Failure	None Reported	Purposive Sampling	Interviews	General Inductive Approach
Jowsey	2009	Australia	Co-morbid chronic illness including DM, COPD and/or CHF	None Reported	Purposive Sampling	Semi-structured in-depth interviews and focus groups	Content Analysis
Keating	2011	Australia	COPD	None reported	Patients who declined or quit participating in a COPD program	Semi-structured interviews	Thematic Analysis
Kirby	2013	Australia	Chronic illness	The Chronic Care Model	Purposive Sampling	Semi-Structured Interviews	Grounded Theory
Kneck	2012	Sweden	Diabetes	None reported	Selective Sampling Approach	Interview	Phenomenological-Hermeneutic Method
Kouwenhoven	2011	Norway	Stroke survivors with early depressive symptoms	None reported	Systematic	Repeated in-depth interviews	Hermeneutic Phenomenology
Kvigne	2004	Norway	Stroke	None reported	Purposive Sampling	Three In-depth Interviews (in hospital, 6-months and 1 year)	Giorgi’s (1985) Phenomenological Four-step < =Method
Loeb	2003	USA	Multiple Chronic Conditions	None Reported	Purposive Sampling	Focus Groups	Thematic and Content Analyses
Lopez-Vargas	2014	Australia	CKD	None Reported	Purposive Sampling	Focus Group	Grounded Theory
Lovely	2013	USA	Malignant Brain Tumor	None Reported	Purposive Sampling	Semi-Structured Interviews	Thematic Analysis
Manias	2007	Australia	Osteoarthritis and at least one other comorbidity	None Reported	Purposive for patients and conveience for health professionals	Focus groups and individual interviews	Framework Analysis
Martini	2012	New Zealand	Gout	None reported	Convenience Sampling	Semi-structured interviews	General Inductive Thematic Approach
Matthias	2010	USA	Chronic Musculoskeletal Pain	None Reported	Purposive Sampling of a subset of participants from previous trial	Focus Groups	Thematic Content Analysis
McCann	2012	USA	Heart disease, diabetes, or osteoporosis	Feminism, Symbolic interactionism. Social networks, social convoy model	Random Sampling Followed by Convenience Sampling	20-min structured telephone interview and 2 face to face follow-up interviews.	Grounded Theory
McCreaddie	2011	UK	Hepatitis C	None Reported	Purposive and, thereafter, Theoretical Sampling	Interviews with patients; focus groups with medical professional staff	Constructivist grounded theory; Constant Comparison
Medina	2011	USA	Chronic disease (included patients had post stroke/diabetes, heart disease/post-TIA, and Parkinson’s/arthritis	The Model of Human Occupation	Purposive Sampling; Key Informants	Two 90-min Face-to-Face Interviews	Phenomenological
Miles	2005	UK	Chronic Pain	None Reported	Theoretical Sampling	Open-ended interviews	Grounded Theory
Mishra	2011	USA	Multiple Chronic Conditions	Bronfenbrenner’s ecological model of behavior	Purposive Sampling	Focus Groups	Phenomenological; Template Analysis
Monroe	2013	USA	HIV AND (diabetes or hypertension)	None reported	Self-referral from flyers and through referral from medical providers	Focus Group	Editing Style Analysis
Morris	2011	UK	Multiple long-term conditions - irritable bowel syndrome; chronic obstructive pulmonary disease; and diabetes	None Reported	Purposive Sampling	Initial face-to-face interviews, telephone follow-ups and final face-to-face interviews	Narrative Analysis
Munce	2014	Canada	Traumatic Spinal Cord Injury	Knowledge to Action framework	Purposive Sampling	Semi-structured telephone interviews	Thematic Analysis
Nakano	2010	USA	Stroke survivor with aphasia	None Reported	Convenience Sampling; single patient case study	In-Depth Interview Over Time	Not Reported
Nelson	2013	UK	Psoriasis	None Reported	Purposive Sampling	Semi-Structured Interviews	Framework Analysis
Newbould	2012	UK	Various chronic conditions	None Reported	Samplingd by voluntary participation from patients from integrated care pilot	Semi-structured Interviews	Lofland and Lofland - Thematic analysis
Newcomb	2010	USA	Asthma	None Reported	Patients were from a trial of asthma care	Semi-Structured Interviews Using a Questionnaire	Constant comparative analysis
Noel	2005	USA	Multiple Chronic Conditions	Von Korff’s Collaborative Management of Chronic Illness Care	Purposive Sampling	Focus Groups	Thematic Analysis
O’Hara	2013	UK	Type 1 Diabetes	None Reported	Self-selected and snowball Sampling	Semi-structured interviews	Grounded Theory
Rifkin	2010	USA	Chronic kidney disease	None Reported	Purposive Sampling	Semi-Structured Interviews	Thematic Analysis
Roberto	2005	USA	Multiple chronic conditions - heart disease, osteoporosis, or diabetes in combination	Life-course Theory and a Trajectory Model of Chronic Illness	Targeted Random Sampling	Semi-Structured Interviews	Thematic Analysis
Rogerson	2012	Australia	CHD and depression	None Reported	Purposively selected from a previous study post-cardiac hospitalization	Semi-structured interviews	Content analysis
Sankar	2003	USA	HIV	None Reported	Targeted and snowball Sampling techniques	Focus Groups	Content and Thematic Analysis
Sav	2013	Australia	Chronic conditions	None Reported	Purposive snowball Sampling	Semi-structured in-depth interviews	Grounded Theory
Schmutte	2009	USA	Serious Mental Illness	None Reported	Participants were recruited through referrals from mental health providers and fliers.	Focus Groups	Interpretive Phenomenological Qualitative Data Analytic Strategies
Schoenberg	2003	USA	Coronary heart disease and risk factors for CHD e.g. hypertension, diabetes, etc.	None Reported	Theoretical Sampling	Interviews and Focus groups	Thematic Analysis
Sells	2009	USA	Multiple chronic conditions	Temporal Framework	Random, stratified Sampling based upon high utilizers vs not	3 Semi-Structured Interviews over 1 year	Phenomenological
Simmonds	2013	UK	Coronary Heart Disease AND Depression	None reported	Consecutive Sampling	Semi-Structured Interview. All the interviews, were digitally recorded, transcribed verbatim	Thematic Analysis
Skuladottir	2011	Iceland	Chronic Pain (women only)	None Reported	Theoretical and Volunteer Sampling	In-depth interviews	Vancouver School of phenomenology
Smith	2012	USA	HIV	The situated Information, Motivation, Behavioral Skills (sIMB) model of Care Initiation and Maintenance for chronic diseases	Purposive Sampling divided between community clinic and medical outreach services	Semi-structured interviews	Content Analysis and Emergent Theme Identification
Snelgrove	2013	UK	Chronic Low Back Pain	IPA and the Enmeshment Model	Purposive Sampling	Semi-Structured Interview	Interpretive Phenomenological Analysis
Söderberg	2001	Sweden	Fibromyalgia	None Reported	Purposive Sampling	Narrative Interviews	Thematic Content Analysis
Soundy	2007	UK	Severe and enduring mental health problems	None Reported	Purposive maximum variation Sampling	Semi-Structured Interview	Thematic Analysis
Taylor	2005	USA	Chronic Fatigue Syndrome	Social Model of Disability	Convenience Sampling	Focus Groups; Open-Ended Questionairre; Progress Notes	Qualitative Comparative Method
Tenhunen	2005	UK	Chronic Daily Headache	None Reported	Purposive Theoretical Sampling; Snowball Sampling	Semi-Structured Interview	Grounded theory
Thompson	2008	USA	Chronic Mental Illness	None Reported	Purposive Sampling	Photovoice and Individual Interview	Qualitative Descriptive Method and Content analysis
Thorpe	2014	Australia	COPD	None Reported	Purposive Sampling	Semi-Structured telephone interviews	Content Analysis
Tollefson	2011	Australia	Chronic Pain	None Reported	Purposive Sampling	Open-ended conversational-type interview	van Manen’s thematic approach
Townsend	2011	Canada	Multimorbidity	Bourdieu’s Theory of Practice	Purposive ﻿Sampling	In-depth interview, a 2-week self-complete symptom/management diary, and a second in-depth interview conducted approximately 3 weeks after the first	Grounded Theory
Treloar	2010	Australia	Hepatitis C and opioid addiction	None Reported	19 randomly Sampling from a larger survey study; 8 recruited from a specific organization	Phone and face to face semi-structured interviews and focus groups	Descriptive analysis
Villena	2010	USA	Mental illness AND substance abuse	None Reported	Purposive Sampling	Semi-Structured Interviews	Interpretive Hermeneutic Phenomenology
Walden	2009	USA	Any chronic condition	None Reported	Purposive Sampling	Individual and Focus Group Interviews; Free Text Survey Comments	Thematic Analysis
Warren-Findlow	2008	USA	Nonobstructive coronary artery disease	None Reported	Purposive Sampling	Multiple In-Depth Interviews conducted over a 2-year time period	Grounded Theory
Wasley	2013	UK	Type 1 diabetes	None Reported	Not reported	Semi-structured interview	Thematic Composition
Webster	2013	Canada	Osteoarthritis	None Reported	Purposive Sampling utilizing maximum variation and Theoretical Sampling	Semi-structured interview.	Constructivist Approach to Grounded Theory
Wendorf	2013	USA	HIV/AIDS and depression	None Reported	Purposive Sampling	Semi-Structured Individual Interviews	Grounded Theory
Wilkinson	2012	UK	Renal Disease	None Reported	Purposive Sampling	Interviews	Thematic Analysis
Williams	2013	Australia	Coexisting Diabetes, CKD and Hypertension	Modified Health Belief Model	Participants in the intervention arm of an RCT, recruited from nephrology and diabetes outpatient clinics	Motivational Interviews conducted via telephone. Data consist of notes taken by the nurse conducting the telephone call	Thematic Analysis
Williams	2013	Australia	Stroke	None Reported	Purposive case Sampling	Semi-structured interviews.	Interpretative Phenomenological Approach
Williams	2009	Australia	Diabetic kidney disease	None Reported	Convenience Sampling	Individual interview	Ritchie and Spencer’s (1994) ‘framework’ method of qualitative analysis
Williams	2008	Australia	Co-exisisting diabetes and kidney disease	Johnson’s (2002) model of medication adherence in hypertensive patients	Convenience Sampling	In-depth interviews and focus groups	Content analysis according to Johnson’s (2002) model of medication adherence in hypertensive patients
Wylde	2011	UK	Chronic Pain (post joint replacement)	None Reported	Convenience Sampling of those who agreed to participate from another survey	Think aloud interviews with existing scale	Thematic Analysis
Wyrwich	2006	USA	Asthma, COPD or heart disease	A model of HRQoL appraisal developed by Rapkin and Schwartz [16]	Theoretical Sampling	Semi-Structured Face-to-Face Cognitive Interviews	Content Analysis
Yang	2009	Australia	Complex Medical conditions - all patients had 2+ comorbidities	None Reported	Clinician referral of patients with 2+ comorbidities 2 weeks after recent hospital discharge	Telephone interviews	Constant Comparative/Grounded Theory
Zanchetta	2007	Canada	Prostate cancer	This enquiry was guided by the philosophy that education is a way to achieve a critical consciousness (Freire, 1973, 1999).	Purposive Sampling	Semi-Structured Interviews, participants’ personal journals, personal documents, genograms and ecomaps, and interviewer’s observational notes	Content Analysis
Zickmund	2012	USA	Hepatitis C and Opioid Addiction	None Reported	Purposive Sampling of patients from a clinical trial	Semi-structured telephone interview	Crabtree and Miller “Editing” Approach

**Table 2:** Methodological rigor of included studies (CASP Checklist)

Study first author last name	Was there a clear statement of the aims of the research?	Is a qualitative methodology appropriate?	Was the research design appropriate to addess the aims of the research?	Was the recruitment strategy appropriate to the aims of the research?	Was the data collected in a way that addressed the research issue?	Has the relationship between researcher and participants been adequately considered?	Have ethical issues been taken into consideration?	Was the data analysis sufficiently rigorous?	Is there a clear statement of findings?
Aspin	Yes	Yes	Can’t tell	Yes	Yes	Can’t tell	Yes	Yes	Yes
Audulv	Yes	Yes	Yes	Yes	Yes	Can’t tell	Yes	Yes	Yes
Bair	Yes	Yes	Yes	Yes	Yes	Can’t tell	Yes	Yes	Yes
Barker	Yes	Yes	Yes	Yes	Yes	Can’t tell	Yes	Yes	Yes
Bayliss	Yes	Yes	Can’t tell	Yes	Yes	Can’t tell	Yes	Yes	Yes
Beauregard	Yes	Yes	Yes	Yes	Yes	Yes	Yes	Yes	Yes
Becker	Yes	Yes	Yes	Yes	Yes	Yes	Yes	Yes	Yes
Becker	Yes	Can’t tell	Can’t tell	Yes	Can’t tell	Yes	Yes	Yes	Yes
Becker	Yes	Yes	Yes	Can’t tell	Yes	Can’t tell	Yes	Yes	Yes
Beverly	Yes	Yes	Can’t tell	Yes	Yes	Can’t tell	Yes	Yes	Yes
Boeckxstaens	Yes	Yes	Yes	Yes	Yes	Can’t tell	Yes	Yes	Yes
Bova	Yes	Yes	Yes	Yes	Yes	Can’t tell	Yes	Yes	Yes
Bower	Yes	Yes	Yes	Yes	Yes	Can’t tell	Yes	Yes	Yes
Bremander	Yes	Yes	Yes	Yes	Yes	Yes	Yes	Yes	Yes
Burles	Yes	Yes	Yes	Yes	Yes	Can’t tell	Yes	Yes	Yes
Carey	Yes	Yes	Yes	Yes	Yes	Yes	Yes	Yes	Yes
Clarke	Yes	Yes	Yes	Yes	Yes	Can’t tell	Yes	Yes	Yes
Corsner	Yes	Yes	Yes	Yes	Yes	Can’t tell	Yes	Yes	Yes
Coty	Yes	Yes	Yes	Yes	Yes	Can’t tell	Yes	Yes	Yes
Drew	Yes	Yes	Yes	Yes	Yes	Can’t tell	Yes	Yes	No
Edmonds	Yes	Yes	Yes	Yes	Yes	Can’t tell	Yes	Yes	Yes
Elliot	Yes	Yes	Can’t tell	Yes	Yes	Can’t tell	Yes	Yes	Yes
Ellis	Yes	Yes	Yes	Yes	Yes	Can’t tell	Yes	Can’t tell	Yes
Eton	Yes	Yes	Yes	Yes	Yes	Can’t tell	Yes	Yes	Yes
Feldman	No	Yes	Yes	Can’t tell	Can’t tell	Yes	Can’t tell	Can’t tell	Yes
Fisher	Yes	Yes	Yes	Yes	Yes	Yes	Yes	Yes	Yes
Foster	Yes	Yes	Yes	Yes	Yes	Can’t tell	Yes	Yes	Yes
Gallant	Yes	Yes	Yes	Yes	Yes	Can’t tell	Can’t tell	Yes	Yes
Garrett	Yes	Yes	Yes	Yes	Yes	Can’t tell	Yes	Yes	Yes
Gelling	Yes	Yes	Yes	Yes	Yes	Can’t tell	Yes	Can’t tell	Yes
Gustaffson	Yes	Yes	Yes	Yes	Yes	Can’t tell	Yes	Yes	Yes
Hodgson	Yes	Yes	Yes	Yes	Yes	Yes	Yes	Yes	Yes
Jakobsen	Yes	Yes	Yes	Yes	Yes	Can’t tell	Can’t tell	Yes	Yes
Janevic	Yes	Yes	Yes	Yes	Yes	Can’t tell	Yes	Yes	Yes
Janke	Yes	Yes	Yes	Yes	Yes	Can’t tell	Yes	Yes	Yes
Janke	Yes	Yes	Yes	Yes	Yes	Can’t tell	Yes	Can’t tell	Yes
Jeon	Yes	Yes	Can’t tell	Yes	Yes	Can’t tell	Yes	Yes	Yes
Jeon	Yes	Yes	Yes	Yes	Yes	Can’t tell	Yes	Yes	Yes
Jeon	Yes	Yes	Yes	Yes	Yes	Can’t tell	Yes	Can’t tell	Yes
Jerant	Yes	Yes	Yes	Yes	Yes	Can’t tell	Yes	Yes	Yes
Jones	Yes	Yes	Yes	Yes	Yes	Yes	Can’t tell	Yes	Yes
Jowsey	Yes	Yes	Can’t tell	Yes	Yes	Can’t tell	Yes	Yes	Yes
Keating	Yes	Yes	Yes	Yes	Yes	Can’t tell	Yes	Yes	Yes
Kirby	Yes	Yes	Yes	Yes	Yes	Can’t tell	Yes	Yes	Yes
Kneck	Yes	Yes	Yes	Yes	Yes	Can’t tell	Yes	Yes	Yes
Kouwenhoven	Yes	Yes	Yes	Yes	Yes	Can’t tell	Yes	Yes	Yes
Kvigne	Yes	Yes	Yes	Yes	Yes	Can’t tell	Yes	Yes	Yes
Liza	Yes	Yes	Yes	Yes	Yes	Yes	Yes	Yes	Yes
Loeb	Yes	Yes	Yes	Yes	Yes	Can’t tell	Yes	Yes	Yes
Lopez-Vargas	Yes	Yes	Yes	Yes	Yes	Can’t tell	Yes	Yes	Yes
Lovely	Yes	Yes	Yes	Yes	Yes	Can’t tell	Yes	Yes	Yes
Manias	Yes	Yes	Yes	Yes	Yes	Can’t tell	Yes	Yes	Yes
Martini	Yes	Yes	Can’t tell	Yes	Yes	Can’t tell	Yes	Can’t tell	Yes
Matthias	Yes	Yes	Yes	Yes	Yes	Can’t tell	Can’t tell	Yes	Yes
McCann	Yes	Yes	Yes	Yes	Yes	Can’t tell	Yes	Yes	Yes
McCreaddie	Yes	Yes	Yes	Yes	Yes	Yes	Yes	Yes	Yes
Medina	Yes	Yes	Yes	Yes	Yes	Can’t tell	Yes	Yes	Yes
Miles	Yes	Yes	Yes	Yes	Yes	Can’t tell	Yes	Yes	Yes
Mishra	Yes	Yes	Yes	Yes	Yes	Can’t tell	Yes	Yes	Yes
Monroe	Yes	Yes	Yes	Yes	Yes	Can’t tell	Yes	Yes	Yes
Morris	Yes	Yes	Yes	Yes	Yes	Can’t tell	Yes	Yes	
Munce	Yes	Yes	Yes	Yes	Yes	Can’t tell	Yes	Yes	Yes
Nakano	Yes	Yes	Can’t tell	Yes	Yes	Yes	Can’t tell	Can’t tell	Yes
Nelson	Yes	Yes	Yes	Yes	Yes	Can’t tell	Yes	Yes	Yes
Newbould	Yes	Yes	Can’t tell	Yes	Yes	Can’t tell	Yes	Yes	Yes
Newcomb	Yes	Yes	No	Yes	No	Can’t tell	Yes	Yes	Yes
Noel	Yes	Yes	Yes	Yes	Yes	Can’t tell	Yes	Yes	Yes
O’Hara	Yes	Yes	Yes	Yes	Yes	Yes	Yes	Yes	Yes
Ohman	Yes	Yes	Yes	Yes	Yes	Can’t tell	Yes	Yes	Yes
Padgett	Yes	Yes	Yes	Yes	Yes	Can’t tell	Yes	Yes	Yes
Paulson	Yes	Yes	Yes	Yes	Yes	Can’t tell	Yes	Yes	Yes
Person	Yes	Yes	Yes	Yes	Yes	Can’t tell	Yes	Yes	Yes
Ravenscroft	Yes	Yes	Yes	Yes	Yes	Can’t tell	Yes	Can’t tell	Yes
Reeve	Yes	Yes	Yes	Yes	Yes	Can’t tell	Yes	Yes	Yes
Riegel	Yes	Yes	Yes	Yes	Yes	Yes	Yes	Yes	Yes
Rifkin	Yes	Yes	Yes	Yes	Yes	Can’t tell	Yes	Yes	Yes
Roberto	Yes	Yes	Yes	Yes	Yes	Can’t tell	Yes	Yes	Yes
Rogerson	Yes	Yes	Yes	Yes	Yes	Can’t tell	Yes	Yes	Yes
Sankar	No	Yes	Yes	Yes	Yes	Can’t tell	Can’t tell	Yes	Yes
Sav	Yes	Yes	Yes	Yes	Yes	Can’t tell	Yes	Yes	Yes
Schmutte	Yes	Yes	Can’t tell	Yes	Yes	Can’t tell	Can’t tell	Yes	Yes
Schoenberg	Yes	Yes	Yes	Yes	Yes	Can’t tell	Yes	Yes	Yes
Sells	Yes	Yes	Yes	Yes	Yes	Can’t tell	Yes	Yes	Yes
Simmonds	Yes	Yes	Yes	Yes	Yes	Can’t tell	Yes	Yes	Yes
Skuladottir	Yes	Yes	Yes	Yes	Yes	Can’t tell	Yes	Yes	Yes
Smith	Yes	Yes	Yes	Yes	Yes	Can’t tell	Yes	Yes	Yes
Snelgrove	Yes	Yes	Yes	Yes	Yes	Yes	Yes	Yes	Yes
Soderberg	Yes	Yes	Yes	Yes	Yes	Can’t tell	Yes	Yes	Yes
Soundy	Yes	Yes	Yes	Yes	Yes	Can’t tell	Yes	Yes	Yes
Taylor	Yes	Yes	Yes	Yes	Yes	Yes	Yes	Yes	Yes
Tenhunen	Yes	Yes	Yes	Yes	Yes	Can’t tell	Can’t tell	Yes	Yes
Thompson	Yes	Yes	Yes	Yes	Yes	Yes	Yes	Yes	Yes
Thorpe	Yes	Yes	Yes	Yes	Yes	Can’t tell	Yes	Yes	Yes
Townsend	Yes	Yes	Yes	Yes	Yes	Can’t tell	Yes	Yes	Yes
Treloar	Yes	Yes	Yes	Yes	Yes	Can’t tell	Yes	Yes	Yes
Walden	Yes	Yes	Yes	Yes	Yes	Yes	Yes	Yes	Yes
Warren-Findlow	Yes	Yes	Yes	Yes	Yes	Yes	Yes	Yes	Yes
Wasley	Yes	Yes	Can’t tell	Can’t tell	No	Can’t tell	Yes	No	No
Webster	Yes	Yes	Yes	Yes	Yes	Yes	Yes	Yes	Yes
Wendorf	Yes	Yes	Yes	Yes	Yes	Can’t tell	Yes	Yes	Yes
Wilkinson	Yes	Yes	Yes	Yes	Yes	Can’t tell	Yes	Yes	Yes
Williams	Yes	Yes	Can’t tell	Yes	No	Can’t tell	Yes	Yes	Yes
Williams	Yes	Yes	Yes	Yes	No	Yes	Yes	Yes	Yes
Williams	Yes	Yes	Yes	Yes	Yes	Can’t tell	Yes	Yes	Yes
Williams	Yes	Yes	Yes	Yes	Yes	Yes	Yes	Yes	Yes
Wylde	Yes	Yes	Yes	Yes	Yes	Can’t tell	Yes	No	Yes
Wyrwich	Yes	Yes	Yes	Yes	Yes	Can’t tell	Yes	Yes	Yes
Yang	Yes	Yes	Yes	Yes	Yes	Can’t tell	Yes	Yes	Yes
Zanchetta	Yes	Yes	Yes	Yes	Yes	Yes	Yes	Yes	Yes
Zickmund	Yes	Yes	Yes	Yes	Yes	Can’t tell	Yes	Yes	Yes

### Major themes

**Fig. 2:**
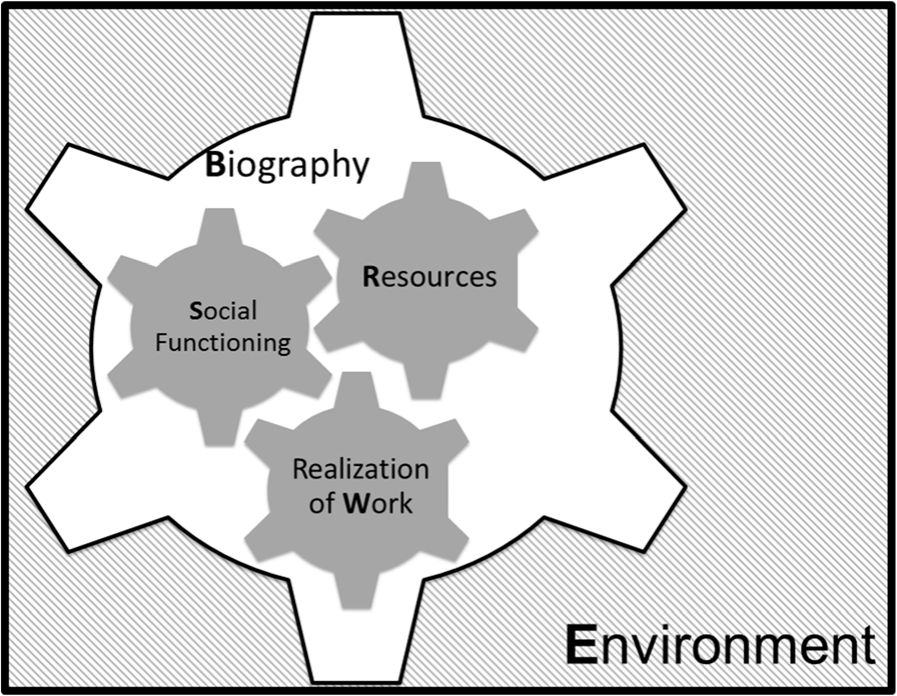
Theory of patient capacity

We found that patient capacity was not simply a set of resources that need to be accessed and mobilized. Instead, it is an accomplishment of interaction, where identifiable psychological and social mechanisms make key contributions. As patients interact with their own biography, resources, environment, patient and life work, and social network, their capacity is either limited or furthered. Also, there were contextual factors that made it easier for patients to cope and self-manage. Figure 2 describes this Theory of Patient Capacity. For each construct, we have provided quotations from included studies.

### Biography

Having chronic disease brought on a host of bothersome symptoms that ultimately disrupted normal life, including employment, housework, and social responsibilities. For some patients, this negatively impacted their quality of life, whereas others were able to reframe and recast their life to account for the new reality.

There was a fundamental difference in the experience of patients who were able to reframe life in the face of chronic disease-i.e., to exercise their ability to make meaning in their lives alongside their chronic conditions -- and those that could not. Reframing could look different for patients with different diagnoses (end-stage renal failure vs. chronic fatigue syndrome) and stages of life (working-age vs. retired), but ultimately, the inclusion of both living with and managing chronic disease and the ability to author his or her vision for life was similarly important. When patients were unable to recreate a new biography that included their illness and its required treatments, they struggled to cope emotionally and to care for their health. They experienced emotional difficulties that they could not overcome, a negative outlook, an inability to cope, lack of control over the situation, and resentment of their conditions. They were at war with their situation.

*“I don’t dedicate myself to anything because I am a sick person. What am I going to dedicate myself to?”* [19].

When people were able to create a new biography that included their illness and treatment, functioning improved. Patients that were successful in their reframing process exercised a host of coping mechanisms that included drawing on spirituality, comparing their situations with others who were worse off, socializing with people who shared or could understand experiences of their condition, engaging in pleasurable activities, meditating, setting out to accomplish tasks, ignoring symptoms altogether, and practicing gratitude.

*“What I’m going to recommend is that you find something that maybe underneath subconsciously has been your passion all your life or is your passion presently…and do something with that passion, if you haven’t already, because I think when you do something you enjoy, that you like, it just takes you to a different level, it takes your mind, it takes your spirit, and you really forget about yourself no matter what that is”* [20].

Reframing one’s biography seemed to color the patient experience; if it occurred, other parts of capacity had the opportunity to function, and if it did not occur, progress was halted. It is unclear if this is causal - the ability to reframe facilitates self-care - or if it is correlational - people who are more able to reframe are also those that will be best at coping with the healthcare tasks they must undertake. Similar concepts have been previously illuminated by Bury, who found that the reframing process during biographical disruption from chronic illness interacted closely with the social networks in which it occurred and the material and cognitive resources available to patients within their environment [21]. Additionally, Charmaz refers to this reframing as changing “identity goals” in the face of chronic illness, which are also dependent upon social context. [22] Corbin and Strauss have pointed out the biographical *work* of the illness experience [23], and Price highlights the need for health professionals to help patients navigate their “illness career”, a process that negotiates the illness experience to discover what is possible and feasible for meaningful living when the illness cannot be cured [24]. Our review highlighted three other interrelated factors that were important in shaping the patient’s capacity: social functioning, resources and their mobilization, and realization of work.

### Social functioning

Social functioning was shaped by the person’s own attributes and their social network. It included the patient’s personal ability to socialize, the ability of their social network to accept the patient’s chronic condition(s) and the changes the condition(s) had caused, the provision of instrumental support, and the social relationships with their healthcare teams.

Some patients were unable to socialize effectively, either due to a pre-existing social disorder or as a consequence of living with a chronic condition. For example, related to a Hepatitis C diagnosis:

*“I developed, from the first time, and still now, a fear of socializing outside with people. And I felt that it really became out of my control, so I literally just completely withdrew. And I was perfectly happy living that way. I really did not want to deal with people, I mean in any way, shape, or form”* [25].

Yet, many patients did not have difficulty with socializing due to any internal limitations of their own, but were faced with unsupportive social networks. For example, some patients encountered family members that did not grasp the different self-management tasks required or colleagues in employment settings that were not sympathetic to symptoms or made it difficult for the patients to undertake the self-management required.

*“All of these supervisors, they want you moving and doing stuff. I’d like to say, ‘Well, my back starts stiffening up or starts aching, I gotta find time to stretch.’ Sometimes they are not real understanding in that”* [26].

The other source of social interaction for patients was the healthcare system. Usually required to understand, receive treatment for, and plan self-management of their condition(s), social interaction in the healthcare system could profoundly affect patient’s capacity to further access and use healthcare and enact self-care. Interactions that negatively affected the patient’s capacity were those in which they were met with disbelief of their experience by the clinician or other healthcare professional, or where they felt not listened and developed distrust for the system.

*“…when I’m trying to talk to them about my problem, and they’ll cut you off. You know, like, ‘You’re not important, you’re wasting my time.’ That's been a real problem for me. It makes you think that no one really cares, especially when it’s done often. It’s not like its 1 or 2 doctors, it’s a lot of them. I have gone to a lot of different doctors”* [27].

*“Well, I can’t tolerate iron and sometimes iron - my doctors kept insisting that I take it and it caused my wrist, hand, and fingers to swell. It was painful, excruciating, but finally by switching types of iron he finally did find one that was good. But for months he would pay no attention to me when I’d tell that. This is what’s doing it. And he’d say - No, it can’t be, it don’t make sense”* [28].

*“They believed that doctors did not understand their condition, did not listen, and at times did not treat them properly. The television producer who experienced cluster headaches said, ‘I’m fighting with these [medical] people to try to get my medicine, and it’s so frustrating.’ He believed that a general practitioner or emergency room physician was not capable of understanding his condition, compared to a specialist such as a neurologist. However, according to the property manager, just because a health care provider is a specialist does not necessarily guarantee that he or she will act in an understanding manner. The property manager described neurologists who did not appear to be truly listening but sat jotting and doodling on their pads. She stated, ‘They treat you [as if] everybody who comes in with a migraine basically is the same; they don’t really listen to you’”* [20].

### Resources

To do work, patients have to mobilize resources in order to access and use healthcare or enact self-care. A resource that patients regularly drew on as part of their experience, and struggled to tap into was physical energy.

*“For example, a lung disease patient stated: ‘… my fatigue… it’s like having energy… I have no energy. Some days I can get up and I… I can start out doing something and an hour later I’m just (wiped-out sound effect), you know, and I try to rest as much as I can in between like housecleaning. I don’t do everything all at once like I used to, for maybe an hour and I sit down and rest and then I’m back up and try, you know, see how much longer I can go, but that’s about it’”* [29].

Other resources that patients tapped into were: time, knowledge, transportation to and from medical appointments or important activities despite of their physical health, physical abilities, finances, paid supportive services, literacy, and self-efficacy.

*“I need it in writing, because I tell you what, I have a lousy memory. And when you’re talking to me over the phone, I don’t usually write all this stuff down”* [30].

*“I find having to buy so much medication is a financial burden even if each medication is cheap. When I have to buy 4 or 5 things a week, it all adds up”* [31].

However, it is important to note that it was not necessarily that the availability of resources *gave* patients capacity, but rather patients had capacity and resources existed in the patient’s lives. What was evident from patient stories was that capacity came from their ability to mobilize new and existing resources and how this enabled them to function in the world. In the example below, the patient was unable to mobilize the rehabilitation program she was resourced with because of her need to use her capacity to care for her child and home. In doing so, she instead needed to draw on her own self-efficacy, physical abilities, and created her own capacity to cope with her disability, a concept discussed in greater detail below (see Buildable Capacity).

*“An ambulant rehabilitation program was planned for her, but it required that she leave home for a few hours 5 days a week, an almost impossible task. After some discussion, her husband got a “sick note” for 2 weeks. At the end of this period, she chose to take over all the tasks and responsibilities for children and home and dropped out of the rehabilitation program. Once home, she had to learn to take care of the baby with a paralysed arm and to use aids in order to accomplish the household tasks. It was hard work, but the importance of caring for her family mobilised her energy: “Sometimes I was totally worn out. The only thing I wanted was to lie down and cry. But one cannot give up. You have to do the things that are needed”* [32].

Similarly, knowledge alone about the patient’s condition(s) in many cases was a resource available in abundance. However, it was patient’s own health literacy, often coming from practical experience, which allowed patients to use that knowledge and facilitated patients’ ability to interact in a productive social manner around their disease, and in turn enact their self-care tasks.

*“I try to eat good healthy food and that I had learned because umm [my husband] was a diabetic for 12 years. So I have learned to eat unsweetened soup and more vegetables, decrease the fat, no cream sauces and I have continued with that”* [33].

Patient self-efficacy was closely related to the ability of patients to use relevant resources to do what they valued. In some cases patients inherently had self-efficacy and in other cases self-efficacy developed from the mastery of tasks learned through their experience with self-care. For example, patients drew on self-efficacy and in some cases self-advocacy, in order to better function in their social environments, which helped them harness their resources, normalize their condition and treatment, and reshape their biography.

*“If I let it get me down, confine me, physically and mentally, then I ain’t gonna be worth nothing. But if I can stay positive about it, I’m doing good. I’m definitely praying about it, constantly. I find praying about it helps me focus on the positive. Even if I fail, I’m still going to try. So if I go out on my bike and I scrub, I’m not gonna give it up”* [27].

### Realization of necessary work

Patients needed to interact with patient and life work. Successfully accomplishing tasks furthered their capacity. In some cases patients simply needed to realize single tasks, while in other cases, they needed to normalize work [34], or make it routine. There is an apparent paradox here: work, for which capacity is needed, can beget capacity. But the cognitive, emotional, and experiential results of successfully completing the work serve to fuel patient capacity. On the other hand, competing life priorities, competing conditions, overwhelming treatment burden, and complex healthcare environments impair doing, sometimes to a point where they are simply too much to allow patients to realize the work set in front of them at a given time. Therefore, the cognitive, emotional, and experiential results of successfully completing that work were absent or replaced by a negative experiential result.

Competing life priorities were related to the patient’s expected life roles, leisure activities, and sometimes connected to cultural norms. Sometimes, patient’s roles were incompatible with patient work, and this created conflict and reduced capacity.

*“Most women are in high stress situations. Most women have children, they take care of the home, they hold down a full time job. Things do not function if the mother’s not there, mother’s never supposed to be sick. She’s always supposed to be there and be able to take care of everybody”* [35].

*“To refuse food, even for health reasons, has implications for the quality of the food served and brings shame on the person offering it. Managing their illnesses by controlling what they ate thus created conflict for many respondents who had diabetes and high blood pressure”* [36].

In the case of competing conditions, patients sometimes attended to a flare of one condition at the expense of the routine for another condition, creating confusion as to what criteria to use to prioritize across conditions.

*“…I’ve had kidney stones about 30 times and every time I get an attack I don’t worry at all about my diet or anything else until I get done treating it to get the pain to go away…I don’t give a single thought to my blood sugar when that happens”* [37].

Sometimes the sheer burden of treatment was too much to normalize.

*“It’s difficult for me because I take 22 pills a day and I take six shots of insulin. That’s just too much medication… It’s too much and, you know, I cry every day because I just don’t feel like taking all those pills”* [38].

And finally, the complexity of the healthcare system hindered the patient’s ability to normalize the condition and treatments in their everyday lives, simply because they were busy navigating the system. This complexity could lead patients to feel as if all their capacity was needed for navigation, rather than other important tasks, whether they were other important healthcare tasks or meaning-making activities.

*“The coordinator is the patient. I felt like I was my own general contractor, marshalling all my subs. A very difficult thing. I’ll say it’s disintegrated health care system. It’s the patient that’s got to make it all happen”* [39].

### Interaction of major themes: a dynamic system of capacity

Most examples provided above were selected to illustrate difficulty with individual components of patient capacity, yet there were many instances of mediation between these social and psychological mechanisms that acted together either for or against the patient’s efforts toward self-care.

For example, struggling with resources sometimes lead to social isolation, which created problems with the patient’s process of reframing their biography.

*“If it’s not one thing it’s the other… Frustrated that I can’t/I was always active. I’m a gregarious person…I feel very isolated because I’m not going anywhere. I don’t have work to go to… it’s driving me up the wall… I’m not meeting people either… I’m not as active as I was. I don’t really go out anywhere… . I can’t like walk from here to the bus stop… either I wouldn’t manage it, or if it was a good day, the time I would get to the bus stop I would be too tired to go anywhere… So it’s taxi, which is 3 down into Paisley and 3 back up again. So, the money side of it holds me back as well…I’m stuck … I just feel my whole life is turned totally turned upside down. … I would like to have more freedom… One money wise and two with my illness…I don’t have the freedom of choice, which is really hard to accept because I’ve always been a person to stand on my own two feet”* [40].

Even in cases where patients could clearly articulate some of the services that they needed access to, they were faced with a lack of empathy. Social interactions like these in healthcare not only caused difficulties for patients in accessing resources and normalizing their condition and treatment, but also permeated these difficulties to the level of the patient biography.

*“In addition to difficulty locating appropriate health care, participants reported problems acquiring disability income, concerns about requesting workplace accommodations, and difficulties accessing community-based resources (such as meal delivery programs and specialized transportation options). These problems occurred because the participants had difficulty convincing their physicians of the need for such resources, because they were unaware of these resources, or because their health care professionals lacked knowledge of how and why they might benefit from such resources.… They faced so much disbelief and negative reactions that they had periods of doubting their own experiences and the legitimacy of their own condition. For this reason they often found it extremely helpful to be in groups of others with CFS. One participant commented, “It’s nice to hear others have similar symptoms and that I am not imagining them all.“ Another participant reported, ”I don’t have to struggle, to my own detriment, to be like everyone else around me that are healthy and inflexible or not interested in educating themselves on CFS. I can find support and understanding”* [41].

Some patients realized and normalized self-care required by their conditions, but compromised their ability to socialize. This was reflected in a dysfunctional reframing process.

*“Now it has passed so long [time], at the beginning it was so clear regarding how much you changed your lifestyle. Now it is more like… now you begin to be more used to it, [you] are a little more withdrawn. Your mood is affected also, you are going to do something and you can’t do everything, then it’s not as fun anymore. You go to the pub and not… yeah… can’t follow the guys in the way you would want to. You go visit a friend and you do bring your syringes, are going to have lunch in town, so you eat your lunch and then some other things happens, maybe you can’t accompany [them] because you haven’t had your snack or maybe not [brought] your dinner insulin or whatever, then it’s just to go home”* [33].

### Facilitating factors

Additionally, patients’ experience illustrated factors that they encountered in their environment, which facilitated the use and development of their capacity to adapt and self-manage including kindness, empathy, and treatment plan fit. When patients encountered kindness and empathy, either in the healthcare system or their own social networks, they were better able to socially function with their illness and treatment, tap into available resources, and normalize the life of being a patient, even in the face of complexity.

*“I wanted someone that at least could treat me like a person … ” Interactions with health care providers where this had occurred were perceived significantly more positively than interactions where it had not. As one participant said: “ … and as a patient that really goes a long way … when you do meet someone that actually shows that kind of care and attention …”* [42].

*“I could miss three visits in 9 months because of my anxiety…walk into the clinic whenever I wanted to, because they know of my anxiety, I’m never denied…they’ll get [any available doctor] when they hear my name, “Oh, shit, she’s here. Let’s get her. I’ve been blessed with them doctors”* [43].

*“Participants also spoke about the importance of patient-provider relationships as a means to help them get through HCV treatment. They discussed the positive aspects of their relationships with various health care providers and how these relationships were integral to evaluation and treatment acceptance. For example, one participant stated, ‘They [doctor and nurse practitioner] explained everything, I was very comforted. I felt taken care of and would give them an A’”* [44].

The fit of the condition, self-care, and healthcare into the patient’s lives helped with the process of realizing healthcare tasks, which meant that patients’ resources were used more effectively, and they were better able to engage in their social settings in a comfortable way. Again this process of successfully realizing work can facilitate cognitive, emotional, and experiential success, to further grown and cultivate the patient’s capacity. Particularly important was health and healthcare that fit the patient’s life did not interfere with competing priorities in life, such as enjoyment time on the weekends where patients were in a different routine. One could imagine treatment plans that were dynamic and were situated in what the patient was engaged in doing at the time.

*“ It’s good when you’re at work, like at work you have routines…and you eat at roughly the same time and take your pills, and at weekends when you’re off all that gets thrown out of the window”* [45].

*“Even though I take walks because I must do it… must do it and because it is good for the diabetes. So I thought that a dog would help. Every day, it would be a couple of times, some longer and some shorter [walks]. It would help with the disease as well”* [33].

*“Finally, some patients described a sense of frustration at the inconvenience of medications, especially with how they interfere with daily life or other important routines like travelling. ‘…that interferes most with my lifestyle. Because of the medication I take, I start taking at 6:00 pm and by 7 pm I’m wasted, just exhausted. I mean it is so.’ ‘Travel is a big issue… making sure I have everything when I go and forgetting something when I get somewhere. It is not real easy to get a prescription transferred’”*.

These examples of both kindness and empathy in healthcare as well as the fit of treatment to the patient’s life, in some respects echo the concept of “person-centered care”, which puts forth that care should 1) afford people dignity, compassion, and respect; 2) offer coordinated care, support, or treatment; 3) offer personalized care, support, or treatment; and 4) support people to recognize and develop their own strengths and abilities to enable them to live an independent and fulfilling life [46].

### Buildable capacity

While patient stories of capacity often discussed the mobilization of *existing* resources or networks, the literature also suggested that capacity could be cultivated and grown. They could not only survive, and cope, but they could also author their own stories. This occurred when patients were able to complete necessary healthcare activities without compromising their pursuit of joy. Activities were intertwined in their lives, and impacted one another. Small victories helped develop greater capacity to continue onward, to reframe their lives, tap into their resources, engage their social networks, and fit their treatment into daily routines. This process furthered their ability to access and use healthcare, adapt, and self-manage.

*“‘I can help others who have been there or who are there. I feel like we’re in a club, so to speak, the ‘Survivor’s Club.’ I can speak to them on a different level than I could Joe on the street because I know what it’s like to be in my position.’ Survivors often found it difficult to reach out to or accept help from others, but doing so brought control to their lives and the opportunity to give something back to others, restoring meaning and value to their lives. Finding activities to keep themselves engaged and active also was important to survivors”* [47].

*“I can do it (make a cup of tea)…hobble… with the crutches into the kitchen pull a wee chair out, sit down, the kettle’s not full. I’ve gotta sit on the chair, turn it round a wee bit, struggle round, hold onto the sink, fill the kettle up, struggle round again, put it back down again. It’s an effort, but it can be done…”* [40].

## Discussion

### Summary of findings

In the body of literature, we found that patient capacity is an accomplishment of interaction with:

1. The processes of reshaping one’s biography in life with chronic condition(s)
2. Resources, their social networks, and the actions required for healthcare and self-care along with and despite of competing priorities
3. An environment of kindness, empathy, and a feasible treatment plan.

### Implications of understanding capacity clinically

In critically thinking about the state of the patient’s capacity, clinicians and other health professionals have a unique opportunity to partner with patients. If clinicians and health professionals seek to respect, support, and build patient capacity for self-management, a shift in thinking is required. Rather than thinking only about the treatment action(s) called for by the clinical discussion at hand by asking “Does this patient have the capacity to do this task?,” they may need to instead consider the action at hand in light of the other capacity-shaping processes going on in the patient’s life by asking, “*How do this patient’s interactions serve to limit or grow their capacity*?” Additionally, they must consider treatment plan fit: *“Does this treatment plan fit with what this patient values doing and being in the world?”* rather than “*Is this treatment plan feasible given their resources?”.*

We have simplified our theoretical framework into a potentially useful mnemonic, to remind busy professionals of the components of capacity that are worthy of consideration beyond resources alone: Biography, Resources, Environment, Work (realization of), and Social (BREWS): *What BREWS patient capacity?* This mnemonic may provide an opportunity to pause in the intensification of treatment if they suspect a patient is struggling with Biographical reframing. By understanding current Resources, they can seek to draw on existing resources instead of those not currently in the patient’s repertoire. Understanding the Environment may illuminate, how the treatment plan fits or doesn’t fit in the patient’s life, or whether the patient has been met by other health professionals with kindness and empathy. If patients are struggling with understanding, realizing, and accepting the Work of *both* patienthood and life, clinicians may refer patients to health or wellness coaches to work with patients to overcome these challenges and create new capacity. Finally, as clinicians understand the patient’s Social interactions with family, friends, and useful others, they may be able to suggest treatment plans that are more socially acceptable to the patient, or connect them with support groups and chronic disease self-management programs. Ultimately, carefully consideration of the patient’s capacity in this way should allow clinicians and other health professionals to work together as a team to design treatment plans that fit their context and support the patient’s capacity to enact this plan.

### Relationship to other literature

Components of the proposed theory can be found in other theories and models as well. However, the effort to synthesize these constructs in light of the Cumulative Complexity Model (CuCoM) and grounded in a wide array of patient experiences, is novel. For example, the concept of reshaping one’s biography in periods of transition in chronic disease is echoed in the Transitions Theory. Characteristics of a healthy transition process include the patient feeling connected, interacting, being situated, and developing confidence and coping [48]. Ultimately, the successful outcome of this process is the mastery of behaviors and skills and an *integrated identity* [48]. The concept of integrated identity, as highlighted above, relates to the concept of biographical construction in the illness experience, which has been explored previously by others [21–24]. Finally, our findings also resonate with the Capabilities Approach when considering patient resources, stating, “that it is not sufficient to know the resources a person owns or can use in order to be able to assess the well-being that he or she has achieved or could achieve; rather, we need to know much more about the person and the circumstances in which he or she is living” [49]. Yet, none of these conceptual frames considers specifically functioning or transition in the face of structural issues relevant to the patient with multiple chronic conditions (i.e., complex and competing clinical guidelines, overwhelming treatment workload, and poorly coordinated healthcare).

This review is supportive of important elements of both the CuCoM and Burden of Treatment Theory (BoT) Theory [50]. The CuCoM postulates that patients have capacity to carry out patient work, which the BoT theory expounds on as defined by their agency, relationality, control, and opportunity to mobilize that capacity. This capacity is critically dependent on the social settings in which it operates, which hinge on the patient’s social skill and social network. Indeed, these constructs bore out in the literature, and remain, in part, in our model of patient capacity. However, our review builds on these to develop a robust, empirically-based Theory of Patient Capacity to inform *capacity building* in these patient groups.

### Limitations

Because we only conducted analysis on reported results from each study, we did not have the opportunity to conduct the original interviews and focus groups, or conduct analysis from original transcripts. We may have missed important elements of the patient stories that were connected to quotes that authors selected to report, or that went beyond the author’s interpretation of the experience. Additionally, as highlighted in our quality appraisal, a handful of included studies had incomplete reporting. This also could have impacted the amount of information presented in the included studies available for analysis. However, because we were able to explore such a breadth of published studies, we gained insight from a much larger sample of patients, with a broad range of conditions, compared to that which we would have encountered in a single, originally-conducted, qualitative study. Additionally, our recoding of the data is inherently connected to our construction of the concepts, which is seen through the lens of the Cumulative Complexity Model and deeply influenced by the thinking of Minimally Disruptive Medicine. However, we sought rigor in our analysis through screening and extracting in duplicate, and also coding a subset of studies in duplicate until we were in agreement, before coding individually.

### Implications for research

There are two areas of needed research that should be considered in light of these findings and the proposed Theory of Patient Capacity. First, future research is needed to explore the constructs that are described within. The qualitative literature suggests that each of these factors play an important role in hindering or furthering the patient’s ability to adapt and self-manage. However, it does little to suggest directionality, order, or magnitude of the associations. Future work should attend to these issues. Additionally, the CuCoM postulates that patient workload-capacity balance affects the extent to which patients are able to access and use healthcare, enact self-care, and their health outcomes. Therefore, associations between constructs described and patients’ success in these areas are worthy of testing. To date, testing the associations between capacity and patient perceived disruption from illness and treatment has to this point focused on the patient’s resources (i.e., physical, emotional, environmental). It would be helpful to understand, for example, what correlation exists between the patient’s biographical reframing (i.e., role function or fulfilment) and their disruption from illness and treatment.

Second, since this research question emerged during the development process for the ICAN Discussion Aid, there is a unique opportunity not often available in the case of conceptual work: the potential for clinicians and health professionals to immediately explore the constructs described here [9]. Health professionals and patients throughout the journey of chronic disease self-management often come to a place in which they feel progress is stalled. Use of the discussion aid and the clinical mnemonic may give glimpse into the areas in which patient capacity is not fully functioning, supporting a partnership that seeks to problem-solve and find a way forward. Using similar tactics with new patients with chronic disease may also pave the way for treatment plans that are in better alignment with patient capacity, and prevent stalling in the future. Future research should attend to making this theory useful in practice.

## Conclusion

As uncovered in this qualitative systematic review, patient capacity is a dynamic accomplishment. The following psychological and social mechanisms hinder or bolster capacity: patient biography, their resources, their environment, their ability to accomplish life and patient work successfully, and their social networks (BREWS). Future research should focus on further exploration of how constructs are related, and of how to apply this theory of capacity in the planning and implementation of treatments in the care of patients with multimorbidity.

## Additional file

Additional file 1: Appendix: Complete Search Strategy for Data. Full Search Strategy used to complete the review. (DOCX 23 kb)

## Abbreviations

BoT: Burden of treatment
BREWS: Biography, Resources, Environment, Work, Social
CASP: Critical appraisal skills programme
CuCoM: Cumulative complexity model
