# Supplementary material for: Patient capacity and constraints in the experience of chronic disease: a qualitative systematic review and thematic synthesis
Source: BMC Fam Pract. 2016 Sep 1;17(1):127. doi: 10.1186/s12875-016-0525-9 (PMC5009523; doi:10.1186/s12875-016-0525-9)
Supplement: Additional file 1: — Appendix: Complete Search Strategy for Data. Full Search Strategy used to complete the review. (DOCX 23 kb) [file 12875_2016_525_MOESM1_ESM.docx]

Appendix: Search Strategies

MEDLINE

1. *kidney failure, chronic/ or *"end-stage renal disease"/ or *"end-stage kidney disease"/ or *esrd/ or *eskd/ or *hemodialysis/ or (kidney failure, chronic or "end-stage renal disease" or "end-stage kidney disease" or esrd or eskd or hemodialysis).ti.

2. exp *diabetes/ or *diabet*/ or (diabetes or diabet*).ti.

3. exp *Heart Failure/

4. 3 or "cardiac failure".ti. or "heart failure".ti.

5. exp *neoplasms/ or cancer*.ti.

6. exp *cognition disorders/ or exp *dementia/ or dement*.ti. or alzheimer*.ti. or (cognitive* adj3 impair*).ti.

7. *hypertension/ or "high blood pressure".ti. or hyperten*.ti.

8. exp *schizophrenia/ or schizophren*.ti.

9. *autism spectrum disorders/ or autism*.ti. or autistic*.ti.

10. exp *Substance-Related Disorders/

11. 10 or (substance adj abuse*).ti. or alcoholi*.ti.

12. exp *Hyperlipidemias/

13. 12 or hyperlipid*.ti. or hypercholesterol*.ti.

14. exp *arrhythmias, cardiac/ or exp *coronary disease/ or exp *myocardial infarction/

15. 14 or coronary.ti. or lvad*.mp. or *heart assist devices/ or "assist device*".mp. or arrhythmia*.ti. or fibrillation*.ti. or tachycardia*.ti. [mp=title, abstract, original title, name of substance word, subject heading word, keyword heading word, protocol supplementary concept word, rare disease supplementary concept word, unique identifier]

16. depress*.ti. or exp *depression/ or exp *depressive disorders/

17. exp *osteoporosis/ or osteopor*.ti.

18. exp *arthritis/ or arthrit*.ti. or osteoarthr*.ti.

19. exp *HIV Infections/

20. 19 or aids.ti. or hiv.ti. or "acquired immunodeficien*".ti.

21. exp *hepatitis/ or hepatitis*.ti.

22. *stroke/ or stroke*.ti. or "cerebrovascular accident*".ti. or ((cerebral or intracranial) adj3 (thrombo* or infarct* or hemorrhag*)).ti.

23. 1 and (2 or 4 or 5 or 6 or 7 or 8 or 9 or 11 or 13 or 15 or 16 or 17 or 18 or 20 or 21 or 22)

24. 2 and (4 or 5 or 6 or 7 or 8 or 9 or 11 or 13 or 15 or 16 or 17 or 18 or 20 or 21 or 22)

25. 4 and (5 or 6 or 7 or 8 or 9 or 11 or 13 or 15 or 16 or 17 or 18 or 20 or 21 or 22)

26. 5 and (6 or 7 or 8 or 9 or 11 or 13 or 15 or 16 or 17 or 18 or 20 or 21 or 22)

27. 6 and (7 or 8 or 9 or 11 or 13 or 15 or 16 or 17 or 18 or 20 or 21 or 22)

28. 7 and (8 or 9 or 11 or 13 or 15 or 16 or 17 or 18 or 20 or 21 or 22)

29. 8 and (9 or 11 or 13 or 15 or 16 or 17 or 18 or 20 or 21 or 22)

30. 9 and (11 or 13 or 15 or 16 or 17 or 18 or 20 or 21 or 22)

31. 11 and (13 or 15 or 16 or 17 or 18 or 20 or 21 or 22)

32. 13 and (15 or 16 or 17 or 18 or 20 or 21 or 22)

33. 15 and (16 or 17 or 18 or 20 or 21 or 22)

34. 16 and (17 or 18 or 20 or 21 or 22)

35. 17 and (18 or 20 or 21 or 22)

36. 18 and (20 or 21 or 22)

37. 20 and (21 or 22)

38. 21 and 22

39. or/28-38

40. (burden* adj2 (treat* or therap*)).mp. [mp=title, abstract, original title, name of substance word, subject heading word, keyword heading word, protocol supplementary concept word, rare disease supplementary concept word, unique identifier]

41. Activities of daily living/ or ADL.mp. or health behavior/ or health, knowledge, attitudes, practice/ or lifestyle.mp. or "life style".mp. or palliat*.mp. or patient education as topic/ or "self care".mp. or "self manage*".mp. or barthel*.mp. or disabilit*.mp. or disabled*.mp. or "work capacity".mp. or "functional disability*".mp. or "functional limit*".mp. or fatigue*.mp. or balance*.mp. or falls.mp. or falling.mp. or severity.mp. or falls, accidental/ or "social support".mp. or supportive.mp. or family.mp. or interpersonal*.mp. or friend*.mp. or companion*.mp. [mp=title, abstract, original title, name of substance word, subject heading word, keyword heading word, protocol supplementary concept word, rare disease supplementary concept word, unique identifier]

42. (Mobility or transportation* or driving or walk* or gait or travel*).mp. or automobile driving/ or mobility limitation/ or tasks.mp. or work*.mp. or "job loss".mp. or "loss of control".mp. or locus of control/ or adaptation*.mp. or unemploy*.mp. or "self esteem".mp. or independen*.mp. or dependent.mp. or "life chang*".mp. or pleasure*.mp. or fear*.mp. or anxious.mp. or anxiety.mp. or leisure*.mp. or medication adherence/ or cope*.mp. [mp=title, abstract, original title, name of substance word, subject heading word, keyword heading word, protocol supplementary concept word, rare disease supplementary concept word, unique identifier]

43. coping.mp. or physician-patient relations/ or communicat*.mp. or partnership.mp. or socioeconomic*.mp. or psychosocial*.mp. or attitude*.mp. or acceptance of health care/ or acceptance.mp. or denial.mp. or choice*.mp. or resilien*.mp. or mobiliz*.mp. or mobilis*.mp. or "social conflict*".mp. or "physical status".mp. or "physical function*".mp. [mp=title, abstract, original title, name of substance word, subject heading word, keyword heading word, protocol supplementary concept word, rare disease supplementary concept word, unique identifier]

44. (Depress* or lonely or loneliness or rehabilitat* or "physical therapy" or stress* or disrupt* or recover* or challeng* or negotiat* or goal* or safe or unsafe or neighborhood or "self efficacy" or social* or isolat*).mp. or social isolation/ or costs.mp. or cost of illness/ or "drug costs".mp. or insurance.mp. or insured.mp. or schedule*.mp. or appointment*.mp. or satisfaction.mp. or satisfy*.mp. or dissatisf*.mp. or suffer*.mp. or delegate*.mp. or qol.mp. [mp=title, abstract, original title, name of substance word, subject heading word, keyword heading word, protocol supplementary concept word, rare disease supplementary concept word, unique identifier]

45. ("quality of life" or "well-being" or dysfunction* or community* or work or intimacy or intimate or emotion* or mood* or unmet or preference* or participat* or "side effect*" or emotion*).mp. [mp=title, abstract, original title, name of substance word, subject heading word, keyword heading word, protocol supplementary concept word, rare disease supplementary concept word, unique identifier]

46. Continuity of patient care/ or accessib*.mp. or needs.mp. or autonomy.mp. or (patient adj2 (care or experience* or understand* or expectation* or perspective* or perception* or satisfaction)).mp. or motivat*.mp. or incentiv*.mp. or disincentive*.mp. or compliant.mp. or noncompliant*.mp. or adheren*.mp. or nonadheren*.mp. [mp=title, abstract, original title, name of substance word, subject heading word, keyword heading word, protocol supplementary concept word, rare disease supplementary concept word, unique identifier]

47. (barrier* or disparit* or language* or education* or experiences or criminal or crime or (low* adj income*) or burden* or disjoint* or access).mp. or health care accessibility/ or distress.mp. or frustrat*.mp. or knowledge*.mp. or beliefs.mp. or expens*.mp. or "out of pocket".mp. or spending.mp. [mp=title, abstract, original title, name of substance word, subject heading word, keyword heading word, protocol supplementary concept word, rare disease supplementary concept word, unique identifier]

48. (multimorbid* or comorbid* or morbidities).mp. [mp=title, abstract, original title, name of substance word, subject heading word, keyword heading word, protocol supplementary concept word, rare disease supplementary concept word, unique identifier]

49. (multiple adj3 (chronic or illness* or morbid* or disease* or condition*)).mp. [mp=title, abstract, original title, name of substance word, subject heading word, keyword heading word, protocol supplementary concept word, rare disease supplementary concept word, unique identifier]

50. ((multi or co) adj morbid*).mp. [mp=title, abstract, original title, name of substance word, subject heading word, keyword heading word, protocol supplementary concept word, rare disease supplementary concept word, unique identifier]

51. "more chronic".mp.

52. or/48-51

53. ("action research" or ethnograph* or ethmolog* or "grounded theory" or (qualitative adj2 (study or studies or data or evidence or synthesis or investigation)) or naturalistic or phenomenolog* or ((semistructured or motivational or structured) adj interview*) or "focus group*" or narrative* or narratolog* or "discourse analysis" or "content analysis" or "constant compar*" or "field stud*" or ((purposive or cluster* or snowball or convenience) adj2 sampl*) or (self adj report*)).mp. [mp=title, abstract, original title, name of substance word, subject heading word, keyword heading word, protocol supplementary concept word, rare disease supplementary concept word, unique identifier]

54. "data saturation".mp. or qualitative research/ or naturalistic.mp. [mp=title, abstract, original title, name of substance word, subject heading word, keyword heading word, protocol supplementary concept word, rare disease supplementary concept word, unique identifier]

55. (audiotap* or videotap* or audiorecord* or videorecord*).mp. [mp=title, abstract, original title, name of substance word, subject heading word, keyword heading word, protocol supplementary concept word, rare disease supplementary concept word, unique identifier]

56. ((audio or video) adj (record* or tape* or taping)).mp. [mp=title, abstract, original title, name of substance word, subject heading word, keyword heading word, protocol supplementary concept word, rare disease supplementary concept word, unique identifier]

57. or/53-56

58. 39 and 57

59. or/40-47

60. 52 and chronic*.mp. [mp=title, abstract, original title, name of substance word, subject heading word, keyword heading word, protocol supplementary concept word, rare disease supplementary concept word, unique identifier]

61. 57 and 60

62. (58 or 61) and (59 or burden*.mp. or complex*.mp. or theme*.mp.) [mp=title, abstract, original title, name of substance word, subject heading word, keyword heading word, protocol supplementary concept word, rare disease supplementary concept word, unique identifier]

63. limit 62 to (english language and yr="2000 - 2014")

64. 52 and 63

EMBASE

1. *kidney failure, chronic/ or *"end-stage renal disease"/ or *"end-stage kidney disease"/ or *esrd/ or *eskd/ or *hemodialysis/ or (kidney failure, chronic or "end-stage renal disease" or "end-stage kidney disease" or esrd or eskd or hemodialysis).ti.

2. exp *diabetes/ or *diabet*/ or (diabetes or diabet*).ti.

3. exp *Heart Failure/

4. 3 or "cardiac failure".ti. or "heart failure".ti.

5. exp *neoplasms/ or cancer*.ti.

6. exp *cognition disorders/ or exp *dementia/ or dement*.ti. or alzheimer*.ti. or (cognitive* adj3 impair*).ti.

7. *hypertension/ or "high blood pressure".ti. or hyperten*.ti.

8. exp *schizophrenia/ or schizophren*.ti.

9. *autism spectrum disorders/ or autism*.ti. or autistic*.ti.

10. exp *Substance-Related Disorders/

11. 10 or (substance adj abuse*).ti. or alcoholi*.ti.

12. exp *Hyperlipidemias/

13. 12 or hyperlipid*.ti. or hypercholesterol*.ti.

14. exp *arrhythmias, cardiac/ or exp *coronary disease/ or exp *myocardial infarction/

15. 14 or coronary.ti. or lvad*.mp. or *heart assist devices/ or "assist device*".mp. or arrhythmia*.ti. or fibrillation*.ti. or tachycardia*.ti. [mp=title, abstract, subject headings, heading word, drug trade name, original title, device manufacturer, drug manufacturer, device trade name, keyword]

16. depress*.ti. or exp *depression/ or exp *depressive disorders/

17. exp *osteoporosis/ or osteopor*.ti.

18. exp *arthritis/ or arthrit*.ti. or osteoarthr*.ti.

19. exp *HIV Infections/

20. 19 or aids.ti. or hiv.ti. or "acquired immunodeficien*".ti.

21. exp *hepatitis/ or hepatitis*.ti.

22. *stroke/ or stroke*.ti. or "cerebrovascular accident*".ti. or ((cerebral or intracranial) adj3 (thrombo* or infarct* or hemorrhag*)).ti.

23. 1 and (2 or 4 or 5 or 6 or 7 or 8 or 9 or 11 or 13 or 15 or 16 or 17 or 18 or 20 or 21 or 22)

24. 2 and (4 or 5 or 6 or 7 or 8 or 9 or 11 or 13 or 15 or 16 or 17 or 18 or 20 or 21 or 22)

25. 4 and (5 or 6 or 7 or 8 or 9 or 11 or 13 or 15 or 16 or 17 or 18 or 20 or 21 or 22)

26. 5 and (6 or 7 or 8 or 9 or 11 or 13 or 15 or 16 or 17 or 18 or 20 or 21 or 22)

27. 6 and (7 or 8 or 9 or 11 or 13 or 15 or 16 or 17 or 18 or 20 or 21 or 22)

28. 7 and (8 or 9 or 11 or 13 or 15 or 16 or 17 or 18 or 20 or 21 or 22)

29. 8 and (9 or 11 or 13 or 15 or 16 or 17 or 18 or 20 or 21 or 22)

30. 9 and (11 or 13 or 15 or 16 or 17 or 18 or 20 or 21 or 22)

31. 11 and (13 or 15 or 16 or 17 or 18 or 20 or 21 or 22)

32. 13 and (15 or 16 or 17 or 18 or 20 or 21 or 22)

33. 15 and (16 or 17 or 18 or 20 or 21 or 22)

34. 16 and (17 or 18 or 20 or 21 or 22)

35. 17 and (18 or 20 or 21 or 22)

36. 18 and (20 or 21 or 22)

37. 20 and (21 or 22)

38. 21 and 22

39. or/28-38

40. (burden* adj2 (treat* or therap*)).mp. [mp=title, abstract, subject headings, heading word, drug trade name, original title, device manufacturer, drug manufacturer, device trade name, keyword]

41. Activities of daily living/ or ADL.mp. or health behavior/ or health, knowledge, attitudes, practice/ or lifestyle.mp. or "life style".mp. or palliat*.mp. or patient education as topic/ or "self care".mp. or "self manage*".mp. or barthel*.mp. or disabilit*.mp. or disabled*.mp. or "work capacity".mp. or "functional disability*".mp. or "functional limit*".mp. or fatigue*.mp. or balance*.mp. or falls.mp. or falling.mp. or severity.mp. or falls, accidental/ or "social support".mp. or supportive.mp. or family.mp. or interpersonal*.mp. or friend*.mp. or companion*.mp. [mp=title, abstract, subject headings, heading word, drug trade name, original title, device manufacturer, drug manufacturer, device trade name, keyword]

42. (Mobility or transportation* or driving or walk* or gait or travel*).mp. or automobile driving/ or mobility limitation/ or tasks.mp. or work*.mp. or "job loss".mp. or "loss of control".mp. or locus of control/ or adaptation*.mp. or unemploy*.mp. or "self esteem".mp. or independen*.mp. or dependent.mp. or "life chang*".mp. or pleasure*.mp. or fear*.mp. or anxious.mp. or anxiety.mp. or leisure*.mp. or medication adherence/ or cope*.mp. [mp=title, abstract, subject headings, heading word, drug trade name, original title, device manufacturer, drug manufacturer, device trade name, keyword]

43. coping.mp. or physician-patient relations/ or communicat*.mp. or partnership.mp. or socioeconomic*.mp. or psychosocial*.mp. or attitude*.mp. or acceptance of health care/ or acceptance.mp. or denial.mp. or choice*.mp. or resilien*.mp. or mobiliz*.mp. or mobilis*.mp. or "social conflict*".mp. or "physical status".mp. or "physical function*".mp. [mp=title, abstract, subject headings, heading word, drug trade name, original title, device manufacturer, drug manufacturer, device trade name, keyword]

44. (Depress* or lonely or loneliness or rehabilitat* or "physical therapy" or stress* or disrupt* or recover* or challeng* or negotiat* or goal* or safe or unsafe or neighborhood or "self efficacy" or social* or isolat*).mp. or social isolation/ or costs.mp. or cost of illness/ or "drug costs".mp. or insurance.mp. or insured.mp. or schedule*.mp. or appointment*.mp. or satisfaction.mp. or satisfy*.mp. or dissatisf*.mp. or suffer*.mp. or delegate*.mp. or qol.mp. [mp=title, abstract, subject headings, heading word, drug trade name, original title, device manufacturer, drug manufacturer, device trade name, keyword]

45. ("quality of life" or "well-being" or dysfunction* or community* or work or intimacy or intimate or emotion* or mood* or unmet or preference* or participat* or "side effect*" or emotion*).mp. [mp=title, abstract, subject headings, heading word, drug trade name, original title, device manufacturer, drug manufacturer, device trade name, keyword]

46. Continuity of patient care/ or accessib*.mp. or needs.mp. or autonomy.mp. or (patient adj2 (care or experience* or understand* or expectation* or perspective* or perception* or satisfaction)).mp. or motivat*.mp. or incentiv*.mp. or disincentive*.mp. or compliant.mp. or noncompliant*.mp. or adheren*.mp. or nonadheren*.mp. [mp=title, abstract, subject headings, heading word, drug trade name, original title, device manufacturer, drug manufacturer, device trade name, keyword]

47. (barrier* or disparit* or language* or education* or experiences or criminal or crime or (low* adj income*) or burden* or disjoint* or access).mp. or health care accessibility/ or distress.mp. or frustrat*.mp. or knowledge*.mp. or beliefs.mp. or expens*.mp. or "out of pocket".mp. or spending.mp. [mp=title, abstract, subject headings, heading word, drug trade name, original title, device manufacturer, drug manufacturer, device trade name, keyword]

48. (multimorbid* or comorbid* or morbidities).mp. [mp=title, abstract, subject headings, heading word, drug trade name, original title, device manufacturer, drug manufacturer, device trade name, keyword]

49. (multiple adj3 (chronic or illness* or morbid* or disease* or condition*)).mp. [mp=title, abstract, subject headings, heading word, drug trade name, original title, device manufacturer, drug manufacturer, device trade name, keyword]

50. ((multi or co) adj morbid*).mp. [mp=title, abstract, subject headings, heading word, drug trade name, original title, device manufacturer, drug manufacturer, device trade name, keyword]

51. "more chronic".mp.

52. or/48-51

53. ("action research" or ethnograph* or ethmolog* or "grounded theory" or (qualitative adj2 (study or studies or data or evidence or synthesis or investigation)) or naturalistic or phenomenolog* or ((semistructured or motivational or structured) adj interview*) or "focus group*" or narrative* or narratolog* or "discourse analysis" or "content analysis" or "constant compar*" or "field stud*" or ((purposive or cluster* or snowball or convenience) adj2 sampl*) or (self adj report*)).mp. [mp=title, abstract, subject headings, heading word, drug trade name, original title, device manufacturer, drug manufacturer, device trade name, keyword]

54. "data saturation".mp. or qualitative research/ or naturalistic.mp. [mp=title, abstract, subject headings, heading word, drug trade name, original title, device manufacturer, drug manufacturer, device trade name, keyword]

55. (audiotap* or videotap* or audiorecord* or videorecord*).mp. [mp=title, abstract, subject headings, heading word, drug trade name, original title, device manufacturer, drug manufacturer, device trade name, keyword]

56. ((audio or video) adj (record* or tape* or taping)).mp. [mp=title, abstract, subject headings, heading word, drug trade name, original title, device manufacturer, drug manufacturer, device trade name, keyword]

57. or/53-56

58. 39 and 57

59. or/40-47

60. 52 and chronic*.mp. [mp=title, abstract, subject headings, heading word, drug trade name, original title, device manufacturer, drug manufacturer, device trade name, keyword]

61. 57 and 60

62. (58 or 61) and (59 or burden*.mp. or complex*.mp. or theme*.mp.) [mp=title, abstract, subject headings, heading word, drug trade name, original title, device manufacturer, drug manufacturer, device trade name, keyword]

63. limit 62 to (english language and yr="2000 - 2014")

64. 52 and 63

65. 64 and (interview* and ("focus group" or qualitative* or sampl* or theme* or thematic*)).mp. [mp=title, abstract, subject headings, heading word, drug trade name, original title, device manufacturer, drug manufacturer, device trade name, keyword]

66. information processing/ or content analysis/ or data synthesis/ or discourse analysis/ or thematic analysis/

67. 64 and 66

68. exp interview/

69. 64 and 68

70. 64 and expectation/

71. 64 and (personal experience/ or patient expectation/)

72. 65 or 67 or 69 or 70 or 71

PsycInfo

1. qualitative research/ or grounded theory/ or interviews/

2. exp Life Experiences/ or lived experience.mp.

3. content analysis/ or discourse analysis/

4. exp Chronic Illness/

5. ((multi or multiple) adj3 (chronic* or morbid*)).mp. [mp=title, abstract, heading word, table of contents, key concepts, original title, tests & measures]

6. exp Comorbidity/ or multimorbid*.mp.

7. or/1-3

8. 7 and (4 or 5 or 6)

9. exp Neoplasms/

10. exp memory disorders/ or exp brain disorders/ or exp cognitive impairment/

11. exp kidney diseases/

12. exp diabetes mellitus/

13. exp heart disorders/

14. exp schizophrenia/

15. exp Autism/

16. exp major depression/

17. exp Osteoporosis/

18. exp Hepatitis/

19. exp Cerebrovascular Accidents/ or exp Cerebral Ischemia/

20. exp hypertension/

21. 9 and (10 or 11 or 12 or 13 or 14 or 15 or 16 or 17 or 18 or 19 or 20)

22. 10 and (11 or 12 or 13 or 14 or 15 or 16 or 17 or 18 or 19 or 20)

23. 11 and (12 or 13 or 14 or 15 or 16 or 17 or 18 or 19 or 20)

24. 12 and (13 or 14 or 15 or 16 or 17 or 18 or 19 or 20)

25. 13 and (14 or 15 or 16 or 17 or 18 or 19 or 20)

26. 14 and (15 or 16 or 17 or 18 or 19 or 20)

27. 15 and (16 or 17 or 18 or 19 or 20)

28. 16 and (17 or 18 or 19 or 20)

29. 17 and (18 or 19 or 20)

30. 18 and (19 or 20)

31. 19 and 20

32. or/21-31

33. 7 and 32

34. 8 or 33

35. limit 34 to (english language and yr="2000 - 2015")

CINAHL

| S18 | S11 AND S15 AND S17 | Limiters - Published Date: 20000101-20141231; English Language; Peer Reviewed; Research Article; Exclude MEDLINE records; Language: English Search modes - Boolean/Phrase | Interface - EBSCOhost Research Databases Search Screen - Advanced Search Database - CINAHL with Full Text | 37 |
| --- | --- | --- | --- | --- |
| S17 | (MH "Patient Compliance+") | Limiters - Published Date: 20000101-20141231; English Language; Peer Reviewed; Research Article; Exclude MEDLINE records; Language: English Search modes - Boolean/Phrase | Interface - EBSCOhost Research Databases Search Screen - Advanced Search Database - CINAHL with Full Text | 1,686 |
| S16 | S11 AND S15 | Limiters - Published Date: 20000101-20141231; English Language; Peer Reviewed; Research Article; Exclude MEDLINE records; Language: English Search modes - Boolean/Phrase | Interface - EBSCOhost Research Databases Search Screen - Advanced Search Database - CINAHL with Full Text | 164 |
| S15 | S12 OR S13 OR S14 | Search modes - Boolean/Phrase | Interface - EBSCOhost Research Databases Search Screen - Advanced Search Database - CINAHL with Full Text | 153,506 |
| S14 | (MH "Health Behavior+") OR (MH "Health Behavior (Iowa NOC) (Non-Cinahl)+") OR (MH "Health Promotion+") OR (MH "Health Seeking Behaviors (NANDA)+") | Search modes - Boolean/Phrase | Interface - EBSCOhost Research Databases Search Screen - Advanced Search Database - CINAHL with Full Text | 73,983 |
| S13 | (MH "Life Style+") OR (MH "Life Style, Sedentary") OR (MH "Life Style Changes") | Search modes - Boolean/Phrase | Interface - EBSCOhost Research Databases Search Screen - Advanced Search Database - CINAHL with Full Text | 100,936 |
| S12 | (MH "Altered Activities of Daily Living (NANDA) (Non-Cinahl)+") OR (MH "Self Care: Activities of Daily Living (Iowa NOC)") OR (MH "Self-Care: Instrumental Activities of Daily Living (Iowa NOC)") OR (MH "Activities of Daily Living (Saba CCC)") OR (MH "Activities of Daily Living Alteration (Saba CCC)") OR (MH "Instrumental Activities of Daily Living (Saba CCC)") OR (MH "Instrumental Activities of Daily Living Alteration (Saba CCC)") OR (MH "Activities of Daily Living+") | Search modes - Boolean/Phrase | Interface - EBSCOhost Research Databases Search Screen - Advanced Search Database - CINAHL with Full Text | 29,478 |
| S11 | S5 AND S10 | Search modes - Boolean/Phrase | Interface - EBSCOhost Research Databases Search Screen - Advanced Search Database - CINAHL with Full Text | 4,619 |
| S10 | S6 OR S7 OR S8 OR S9 | Search modes - Boolean/Phrase | Interface - EBSCOhost Research Databases Search Screen - Advanced Search Database - CINAHL with Full Text | 53,065 |
| S9 | (MH "Chronic Disease") | Limiters - Published Date: 20000101-20141231 Search modes - Boolean/Phrase | Interface - EBSCOhost Research Databases Search Screen - Advanced Search Database - CINAHL with Full Text | 24,451 |
| S8 | multimorbid* OR comorbid* OR "co-occurring" | Limiters - Published Date: 20000101-20141231 Search modes - Boolean/Phrase | Interface - EBSCOhost Research Databases Search Screen - Advanced Search Database - CINAHL with Full Text | 29,963 |
| S7 | (MH "Comorbidity") | Limiters - Published Date: 20000101-20141231 Search modes - Boolean/Phrase | Interface - EBSCOhost Research Databases Search Screen - Advanced Search Database - CINAHL with Full Text | 20,417 |
| S6 | "multimorbidity" | Limiters - Published Date: 20000101-20141231 Search modes - Boolean/Phrase | Interface - EBSCOhost Research Databases Search Screen - Advanced Search Database - CINAHL with Full Text | 164 |
| S5 | S1 OR S2 OR S3 | Search modes - Boolean/Phrase | Interface - EBSCOhost Research Databases Search Screen - Advanced Search Database - CINAHL with Full Text | 152,124 |
| S4 | (MH "Cancer Patients") OR (MH "Cardiac Patients") | Search modes - Boolean/Phrase | Interface - EBSCOhost Research Databases Search Screen - Advanced Search Database - CINAHL with Full Text | 20,726 |
| S3 | (MH "Thematic Analysis") OR (MH "Semantic Analysis") OR (MH "Content Analysis") OR (MH "Constant Comparative Method") | Search modes - Boolean/Phrase | Interface - EBSCOhost Research Databases Search Screen - Advanced Search Database - CINAHL with Full Text | 44,547 |
| S2 | (MH "Qualitative Studies+") | Search modes - Boolean/Phrase | Interface - EBSCOhost Research Databases Search Screen - Advanced Search Database - CINAHL with Full Text | 68,311 |
| S1 | (MH "Interviews+") | Search modes - Boolean/Phrase | Interface - EBSCOhost Research Databases Search Screen - Advanced Search Database - CINAHL with Full Text | 118,309 |
